# Endoscopic purse-string suture combined with metal clips for the closure of a large gastro-pleural fistula

**DOI:** 10.1055/a-2721-9564

**Published:** 2025-11-04

**Authors:** Xinhui Li, Wenming Wu, Jing Wang, Xiaofeng Liu

**Affiliations:** 1 707693 Department of Gastroenterology, The 960th Hospital of PLA, Jinan, China; 2 518873 Shandong First Medical University, Jinan, China

A 76-year-old female patient, more than two decades post-esophagectomy for esophageal cancer, presented with recurrent episodes of melena. Gastroscopic examination revealed ulceration in the remnant stomach, and the initial management was conservative, involving medication. The patient was subsequently admitted due to episodes of vomiting material resembling coffee grounds. An upper gastrointestinal contrast study identified a fistula tract along the lesser curvature of the gastric body, with the evidence of contrast extravasation ( Fig. 1 ). A chest computed tomography scan revealed a linear contrast shadow posterior to the left heart ( Fig. 2 ). Endoscopic evaluation identified a fistula with regular edges, approximately 10 mm in diameter, located on the lesser curvature of the mid-gastric body, through which pericardial pulsations within the thoracic cavity were observable. The mucosal edges were treated with argon plasma coagulation (APC), and the fistula was successfully closed using a purse-string suture technique, facilitated by metal clips and a nylon loop ( Fig. 3 ). On postoperative day 1, contrast imaging confirmed complete closure with well-defined edges ( Fig. 4 ). At a 3-month follow-up conducted via telephone, the patient reported no recurrence of hematemesis or melena and experienced no significant discomfort, but declined to undergo repeat endoscopy.

**Fig. 1:**
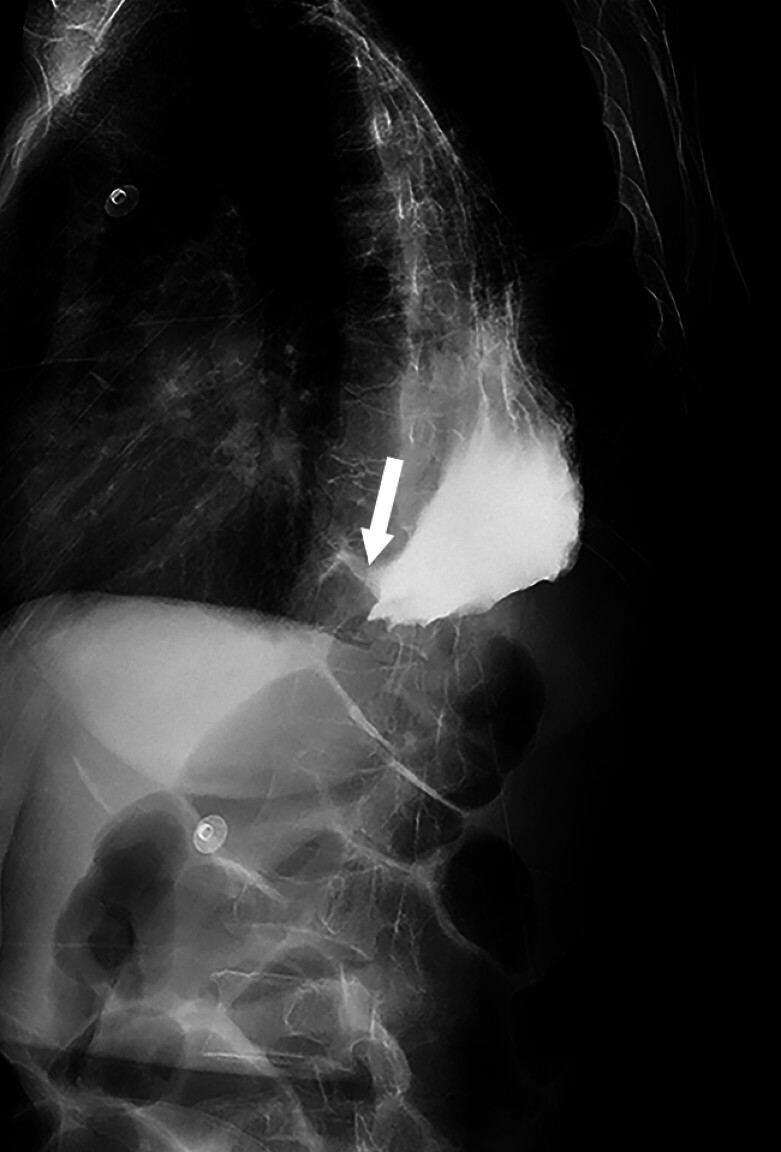
An upper gastrointestinal contrast study demonstrated contrast extravasation.

**Fig. 2:**
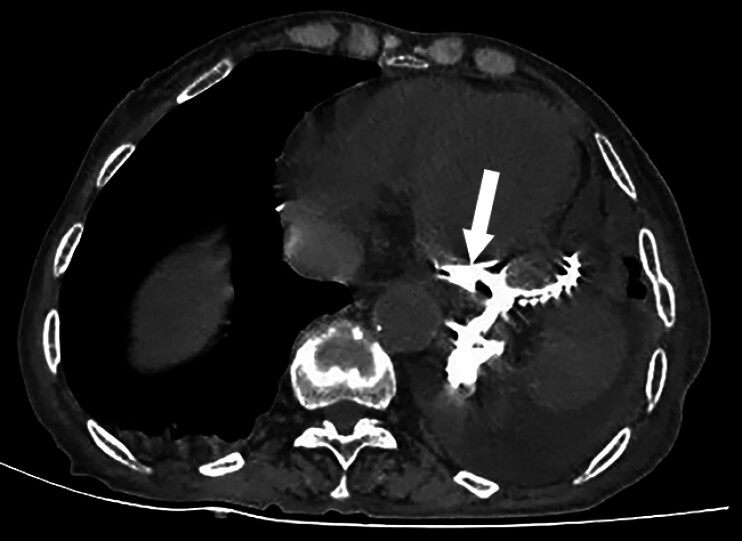
A chest computed tomography scan revealed a linear contrast shadow posterior to the left heart.

**Fig. 3:**
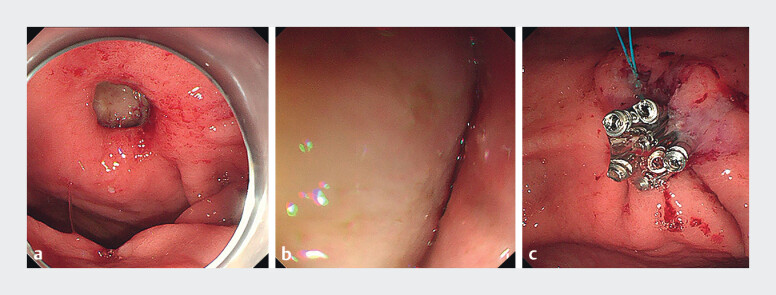
Endoscopic intervention was performed to close the fistula using a combination of metal clips and a purse-string suture. **a** Endoscopic identification of a fistula approximately 10 mm in diameter. **b** Pericardial pulsation within the thoracic cavity. **c** Successful fistula closure using the combined approach of metal clips and a purse-string suture.

**Fig. 4:**
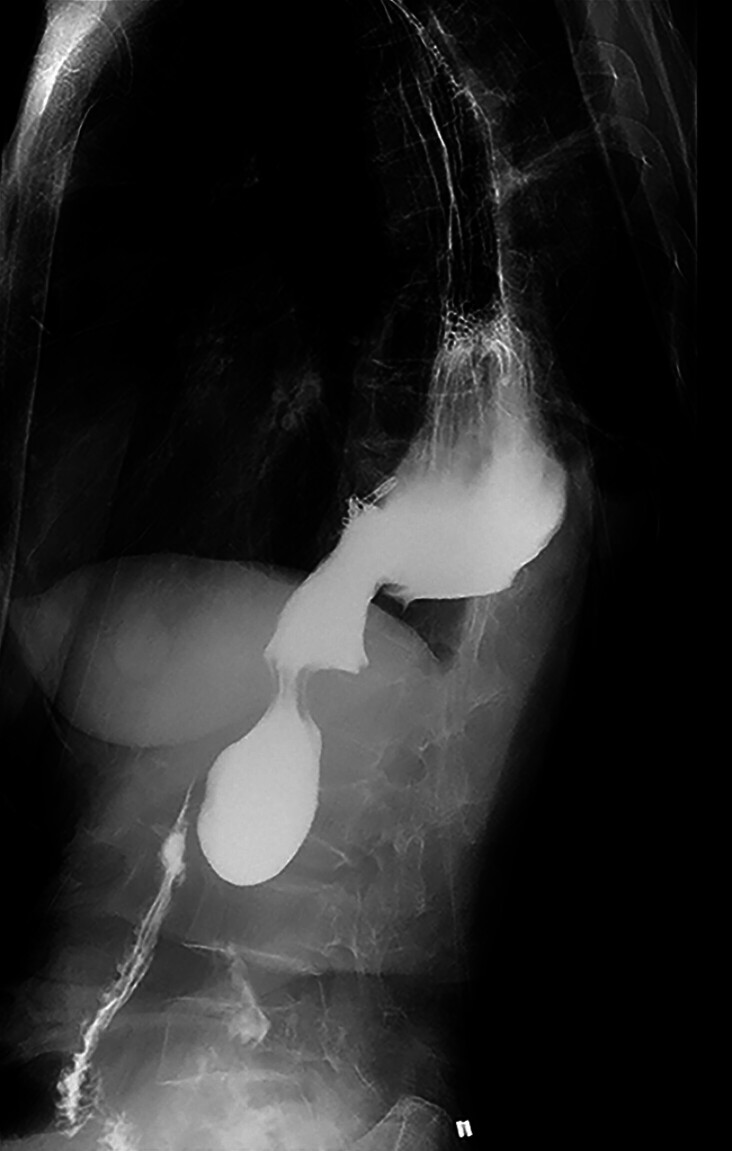
A follow-up upper gastrointestinal contrast study indicated the absence of contrast extravasation.

Gastric fistulas are infrequent occurrences that may result in complications such as pain, mediastinitis, sepsis, organ failure, or mortality 1 . Standard therapeutic approaches typically involve endoscopic and surgical interventions 2 . The development of a gastro-pleural fistula following esophagectomy, attributed to anatomical alterations, is exceedingly rare. This case report documents the inaugural successful closure of such a fistula utilizing APC in conjunction with an endoscopic purse-string suture, supported by metal clips and a nylon loop, ultimately achieving complete fistula closure and therapeutic success ( Video 1 ).

Endoscopic purse-string suture combined with metal clips for the closure of a large gastro-pleural fistula.Video 1

Endoscopy_UCTN_Code_TTT_1AO_2AI

## Notes

Endoscopy E-Videos https://eref.thieme.de/e-videos
*E-Videos* is an open access online section of the journal *Endoscopy* , reporting on interesting cases and new techniques in gastroenterological endoscopy. All papers include a high-quality video and are published with a Creative Commons CC-BY license. Endoscopy E-Videos qualify for HINARI discounts and waivers and eligibility is automatically checked during the submission process. We grant 100% waivers to articles whose corresponding authors are based in Group A countries and 50% waivers to those who are based in Group B countries as classified by Research4Life (see: https://www.research4life.org/access/eligibility/ ). This section has its own submission website at https://mc.manuscriptcentral.com/e-videos .
